# Acute haemorrhagic conjunctivitis outbreak attributed to coxsackievirus A24 in Ratanakiri, Cambodia, 2023

**DOI:** 10.5365/wpsar.2026.17.1.1226

**Published:** 2026-02-23

**Authors:** Kimhour Lay, Kossama Chukmol, Guechlaing Chea, Leng Un, Kimhong Moch, Seiha Do, Lykheang Lou, Meng Ngy, Piseth Kong

**Affiliations:** a Preah Ang Duong Hospital, Phnom Penh, Cambodia.; b Cambodian Ophthalmological Society, Phnom Penh, Cambodia.; c Provincial Referral Hospital of Ratanakiri, Ratanakiri, Cambodia.; d National Program for Eye Health, Phnom Penh, Cambodia.; e Khmer–Soviet Friendship Hospital, Phnom Penh, Cambodia.

## Abstract

**Objective:**

To determine the causative agent, clinical manifestations and risk factors for infection during a September 2023 outbreak of acute haemorrhagic conjunctivitis (AHC) in Pak Touch village, Ratanakiri province, Cambodia.

**Methods:**

A retrospective case-control study was conducted. Cases were age-matched to controls (1:1), who were randomly selected from the village population. Twenty-one conjunctival samples were analysed using real-time reverse transcription–polymerase chain reaction (RT–PCR). RNA sequencing was additionally performed to identify the causative agent of the outbreak. Logistic regression models were used to identify significant risk factors.

**Results:**

A total of 73 cases and 73 controls were included in the analysis. Cases had a median age of 20 years (range: 1–70, mean and standard deviation: 27.7 ± 20.0), and 46.6% (34/73) were male. The overall attack rate was 12.3% (73 cases/594 residents). Clinical presentations included conjunctival hyperaemia (100%), subconjunctival haemorrhage (82.2%, 60), pain and discharge (64.4%, 47 each), eyelid swelling (57.5%, 42) and tearing (54.8%, 40). RT–PCR identified enterovirus in 52.4% (11/21) of conjunctival swabs, with RNA sequencing confirming the coxsackievirus A24 variant as the causative agent in five swabs. Statistical analysis identified significant risk factors, including physical contact with patients with acute haemorrhagic conjunctivitis (adjusted odds ratio [aOR]: 4.42, 95% confidence interval [CI]: 1.90–10.10), frequent eye rubbing (aOR: 4.56, 95% CI: 2.00–10.37) and poor hand hygiene (aOR: 3.70, 95% CI: 1.64–8.43).

**Discussion:**

The outbreak of acute haemorrhagic conjunctivitis in Pak Touch village was primarily caused by coxsackievirus A24. Significant risk factors included physical contact with infected individuals, frequent eye rubbing and poor hand hygiene. Effective hygiene measures are crucial to prevent the spread of AHC.

Acute haemorrhagic conjunctivitis (AHC) was initially reported in Ghana in 1969 and rapidly spread across Africa, South-East Asia and Japan. (*1*) It was nicknamed “Apollo 11 disease” due to its emergence coinciding with the Apollo 11 moon landing. (*2*) This highly contagious viral disease is characterized by the sudden onset of a red eye, eyelid swelling, tearing and subconjunctival haemorrhage. These symptoms typically appear after an incubation period of 12–48 hours and resolve spontaneously within 1–2 weeks. While AHC is not usually a serious condition, it can cause discomfort and disrupt daily life, and in rare cases it can lead to ocular and systemic complications. (*3*-*5*)

Outbreaks of AHC are often linked to enteroviruses, particularly *Enterovirus D 70* (EV-D70) and coxsackievirus A24 variant (CV-A24v), along with certain adenovirus strains. (*6*-*10*) EV-D70 was responsible for pandemics from the early 1970s to the mid-1980s, but its role in causing AHC declined significantly after 1994. (*3*, *11*-*13*) CV-A24v, a variant of the Joseph prototype strain within the *Enterovirus C* species first isolated in Singapore in 1970, has since gained recognition as the leading cause of AHC outbreaks worldwide. (*14*-*16*)

AHC spreads mainly through direct contact with contaminated fingers, objects and tears. However, in the case of CV-A24v – which uses sialic acids as receptors, binding α2,3 on the cornea and α2,6 in the respiratory tract – transmission also occurs via airborne secretions. (*17*, *18*) Factors such as crowded environments and poor hygiene, and practices such as sharing towels and reusing bathing water further accelerate transmission. (*19*)

Rapid diagnosis and virological confirmation are essential for differentiating AHC from other eye infections and triggering appropriate treatment and control strategies to prevent epidemics. However, virus identification remains a challenging and time-consuming process. Initial isolation from conjunctival samples involves inoculation onto human cell lines (MRC5 or Hep-2) to detect cytopathic effects, with specificity confirmed using real-time reverse transcription–polymerase chain reaction (RT–PCR), immunofluorescence or seroneutralization assays. (*11*, *20*, *21*) However, CV-A24v cannot be identified through these conventional methods, necessitating *VP1* sequencing, which requires several days to yield results. (*22*-*25*) While effective, these methods still face sensitivity challenges and high costs, limiting accessibility in developing countries.

In Cambodia, the first major outbreak of AHC was reported in July 1980 and was attributed to EV-D70. It occurred 1 year after the fall of the Khmer Rouge, when the disease was prevalent in refugee camps and designated transit centres. (*26*) Since then, Cambodia had not had any reported cases of AHC. However, on 23 September 2023, the director of Soam Thom Health Center reported a surge in cases of conjunctivitis in a village in Ratanakiri province. On 24 September, the Communicable Disease Control Department of the Cambodian Ministry of Health assigned a rapid response team to investigate the outbreak, which was later confirmed to be AHC. The outbreak was linked to four cases occurring among eight individuals who had recently returned to Pak Touch village from a funeral in Viet Nam on 14 September 2023. A public health education campaign was promptly initiated by the Ministry of Health and the National Program for Eye Health to reduce further spread.

A retrospective study was conducted using data collected during the investigation of the outbreak, and this study aimed to characterize the AHC epidemic in Pak Touch village, Ratanakiri province, Cambodia. The objectives were to describe the clinical manifestations and to identify the causative agent and risk factors for infection.

## Methods

### Study design and setting

This retrospective study used a case-control design. Pak Touch village is situated in Pak Nhai commune, O’yadav district, Ratanakiri province. It is 23 km from Soam Thom Health Center, 73 km from Ban Lung City, and 15 km from the Viet Nam border. The village is predominantly inhabited by people of the Charay ethnic group; the village faces challenges related to hygiene and sanitation, (*27*) and farming is the main occupation. It is home to 164 families, comprising a total population of 594 residents, including 297 males and 297 females.

### Outbreak investigation

**Fig. 1:**
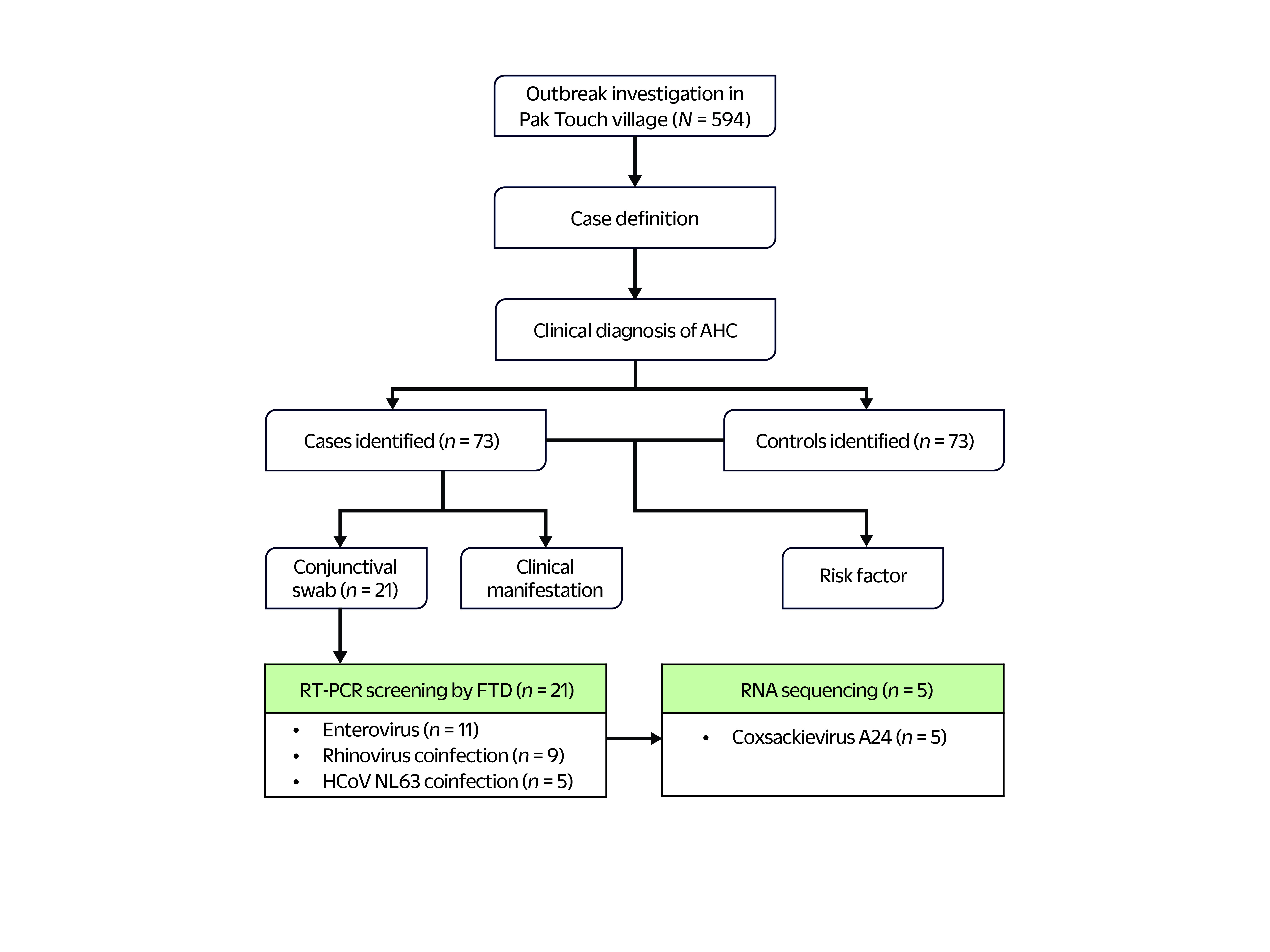
Schematic of the investigation of the outbreak of acute haemorrhagic conjunctivitis in Pak Touch village, Ratanakiri province, Cambodia, 2023

The initial investigation started on 24 September 2023, following a report from the director of the Soam Thom Health Center about a significant rise in the number of cases of acute conjunctivitis. The investigation protocol is illustrated in **Fig. 1**. The following case definitions were employed:

[**insert** Figure 1]

suspected AHC case – any individual presenting with eye redness, eye pain or eyelid swelling on or after 14 September 2023, in Pak Touch village;
confirmed AHC case – any clinical case of conjunctivitis that was diagnosed by an ophthalmologist and characterized by eye redness, eye pain, eyelid swelling and/or subconjunctival haemorrhage, occurring on or after 14 September 2023, in Pak Touch village.

### Identification of cases and controls

For this case-control study, cases were identified through a review of medical records and the line lists compiled by local health authorities on 24 September 2023, the day of the investigation. Individuals who had been clinically confirmed as having AHC by an ophthalmologist on 24 September 2023 were included in the study as cases. Patients diagnosed with conjunctivitis before 14 September 2023 were excluded from the case-control analysis. Each case was matched to a control of the same age (i.e. year of birth ± 2 years) selected at random from the population registry for the village. Potential controls were excluded if their medical records were incomplete or missing.

### Definitions of risk factor variables

Demographic data (age and sex) were obtained from medical records, and data about the date of symptom onset among cases and potential risk factors were obtained from records of face-to-face interviews conducted during the field investigation by the response team. Nine risk factor variables were included in the analysis: having an AHC patient in the family, meeting with an AHC patient, having physical contact with an AHC patient, sharing eye drops, using the same toilet, rubbing eyes frequently, drinking from the same source (e.g. sharing cups), sharing a towel and having poor hand hygiene. Poor hand hygiene was defined as the absence of regular handwashing with soap and water, particularly before eating and after using the toilet.

### Laboratory investigation

#### Sample collection

During the initial investigation, conjunctival swabs were collected from 21 patients with clinical signs of AHC. After aseptic collection, specimens were placed in test tubes containing 2 mL of minimal essential medium, stored at 4 °C and transported to the National Public Health Laboratory of Cambodia. The cold chain was maintained for 24 hours for further analysis. Real-time RT–PCR was used to identify the viral pathogen; its genomic properties were identified using RNA sequencing.

#### Testing for respiratory pathogens

Real-time RT–PCR testing was conducted at the National Public Health Laboratory using Fast Track Diagnostics’ Respiratory Pathogens 21 multiplex assay (Fast Track Diagnostics, Esch-sur-Alzette, Luxembourg), designed to identify 20 viruses and one bacterium.

#### RNA sequencing

To confirm the causative agent of the outbreak, the samples underwent comprehensive genomic sequencing using the Illumina RNA Prep with Enrichment tagmentation kit for targeted RNA sequencing, according to the manufacturer’s instructions (Illumina, San Diego, CA, USA). The sequencing results were analysed using CZ ID, a cloud-based platform (Chan Zuckerberg Initiative).

### Statistical analysis

For this case-control study, the characteristics of cases were described using frequencies and percentages for categorical variables, and the median and range, and mean and standard deviation (SD) for continuous variables (age). The attack rate was calculated using the following formula:

where the population at risk was 594, the number of people living in Pak Touch.

χ^2^ tests were performed to compare the distribution of categorical variables between cases and controls. Simple logistic regression was used to estimate the association between an individual risk factor variable and AHC infection, with results presented as crude odds ratios (cORs) and corresponding 95% confidence intervals (CIs). Multiple logistic regression was then conducted to identify factors independently associated with the outcome after adjusting for potential confounders. Final model results are reported as adjusted odds ratios (aORs) with 95% CIs and corresponding *P*-values. Data analysis was conducted using STATA version 18 (StataCorp, College Station, TX, USA).

## Results

### Demographic characteristics and risk factor prevalence

On the date of the investigation, 73 cases were identified, representing an attack rate of 12.3%. For the risk factor analysis, 73 controls were subsequently recruited. The median age of cases was 20.0 years (range: 1–70, mean ± SD: 27.7 ± 20.0). Due to age-matching, the age distribution between cases and controls was not statistically significant. Nor was there a significant difference in the sex distribution between cases and controls (cases: 53.4% female, 39/73; controls: 52.1% female, 38/73; *P* = 0.868) (**Table 1**).

**Table 1:** Sociodemographic characteristics of cases of acute haemorrhagic conjunctivitis and controls (*n* = 146), Pak Touch village, Cambodia, 2023

Characteristic	Cases (*n* = 73)	Controls (*n* = 73)	Χ^2^	*P*
**Demographic variables**				
**Median age (range, mean ± SD)**	**20.00 (1–70, 27.7 ± 20.0)**	**22.00 (2–70, 29.4 ± 19.9)**	**-**	**-**
**Age group (years)**				
** < 6**	**7 (9.6)**	**4 (5.5)**	**-**	**-**
**6–18**	**19 (26.0)**	**31 (42.5)**	**-**	**-**
**19–30**	**13 (17.8)**	**11 (15.1)**	**-**	**-**
**31–50**	**19 (26.0)**	**16 (21.9)**	**-**	**-**
** ≥ 51**	**15 (20.5)**	**11 (15.1)**	**-**	**-**
**Sex**				
**Male**	**34 (46.6)**	**35 (47.9)**	**-**	**-**
**Female**	**39 (53.4)**	**38 (52.1)**	**0.0275**	**0.868**
**Risk factors**	**-**	**-**	**-**	**-**
**Met with AHC patient**				
**No**	**22 (30.1)**	**47 (64.4)**	**-**	**-**
**Yes**	**51 (69.9)**	**26 (35.6)**	**17.1749**	** < 0.001**
**Physical contact with AHC patient**				
**No**	**16 (21.9)**	**50 (68.5)**	**-**	**-**
**Yes**	**57 (78.1)**	**23 (31.5)**	**31.9652**	** < 0.001**
**Had AHC patient in family**				
**No**	**31 (42.5)**	**52 (71.2)**	**-**	**-**
**Yes**	**42 (57.5)**	**21 (28.8)**	**12.3133**	** < 0.001**
**Used the same toilet**				
**No**	**29 (39.7)**	**35 (48.0)**	**-**	**-**
**Yes**	**44 (60.3)**	**38 (52.0)**	**1.0015**	**0.317**
**Shared eye drops**				
**No**	**50 (68.5)**	**63 (86.3)**	**-**	**-**
**Yes**	**23 (31.5)**	**10 (13.7)**	**6.6168**	**0.001**
**Shared cups for drinking**				
**No**	**27 (37.0)**	**46 (63.0)**	**-**	**-**
**Yes**	**46 (63.0)**	**27 (37.0)**	**9.8904**	**0.002**
**Shared towel**				
**No**	**48 (65.8)**	**54 (74.0)**	**-**	**-**
**Yes**	**25 (34.2)**	**19 (26.0)**	**1.1711**	**0.279**
**Rubbed eyes frequently**				
**No**	**18 (24.7)**	**50 (68.5)**	**-**	**-**
**Yes**	**55 (75.3)**	**23 (31.5)**	**28.1870**	** < 0.001**
**Poor hand hygiene**				
**No**	**20 (27.4)**	**48 (65.8)**	**-**	**-**
**Yes**	**53 (72.6)**	**25 (34.2)**	**21.5807**	** < 0.001**

AHC: acute haemorrhagic conjunctivitis; SD: standard deviation; χ^2^: χ^2^ test.

Values are *n*(%) unless otherwise indicated. Bold *P*-values are statistically significant (< 0.05).

Cases and controls differed in terms of the proportion who reported having met with AHC patients (69.9% [51/73] for cases vs 35.6% [26/73] for controls, *P* < 0.001) and who had physical contact with AHC patients (78.1% [57] vs 31.5% [23], *P* < 0.001) (**Table 1**). Having an AHC patient in the family was also more common among cases than controls (57.5% [42] vs 28.8% [21], *P* < 0.001). The prevalence of risk factors related to hygiene and sharing practices was also higher in cases than in controls. A higher proportion of cases reported sharing eye drops (31.5% [23] vs 13.7% [10], *P* = 0.001); drinking from the same cup was reported by 63.0% (46) of cases but only 37.0% (27) of controls (*P* = 0.002). Frequent eye rubbing was more common among cases (75.3% [55] vs 31.5% [23], *P* < 0.001), as was poor hand hygiene (72.6% [53] vs 34.2% [25], *P* < 0.001).

### Clinical presentation

Conjunctival hyperaemia was the most common clinical manifestation of AHC, observed in all 73 cases (100%). Subconjunctival haemorrhage was experienced by 82.2% (60/73), while pain and discharge were each reported by 64.4% (47/73 each). Eyelid swelling was observed in 57.5% (42/73) and tearing in 54.8% (40/73) (**Table 2**). During the investigation, none of the patients developed systemic manifestations or subsequent complications.

**Table 2:** Clinical presentation of cases with acute haemorrhagic conjunctivitis, Pak Touch village, Cambodia, 2023 (*n* = 73)

Clinical presentation	No. (%) of cases
**Eyelid swelling**	**42 (57.5)**
**Discharge**	**47 (64.4)**
**Pain**	**47 (64.4)**
**Tearing**	**40 (54.8)**
**Conjunctival hyperaemia**	**73 (100.0)**
**Subconjunctival haemorrhage**	**60 (82.2)**

**Fig. 2:**
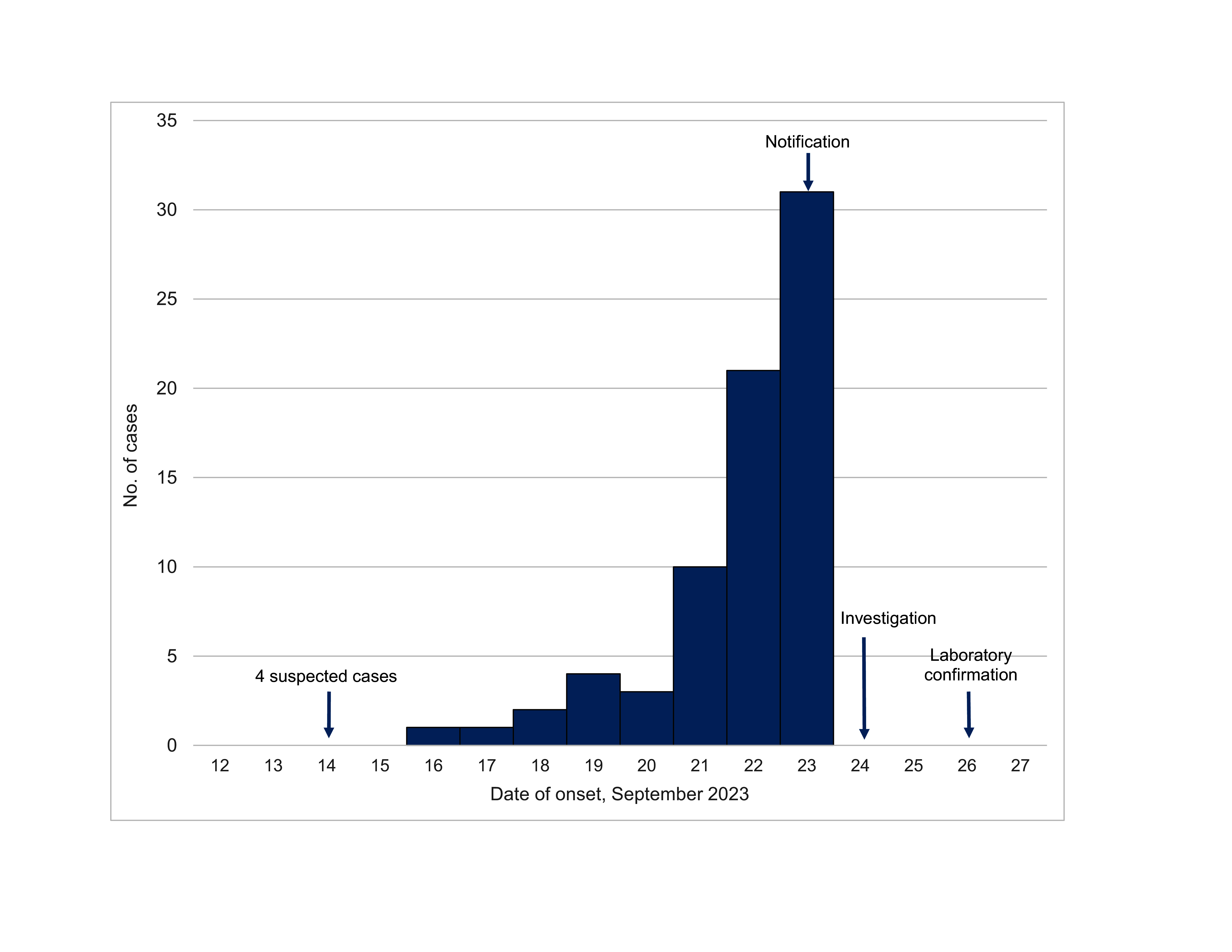
Epidemic curve of outbreak of acute haemorrhagic conjunctivitis in Pak Touch village (N = 73), Ratanakiri province, Cambodia, 14–24 September 2023, based on the first day of clinical presentation as reported by the patient

The propagated epidemic curve, shown in **Fig. 2**, revealed a steady rise in the number of individuals presenting with symptoms between 16 and 23 September 2023. The earliest case presentation, on 16 September 2023, occurred 2 days after the individuals returned from Viet Nam. Reported case numbers increased thereafter, peaking at 31 new cases on 23 September 2023.

[**insert** Figure 2]

### Risk factor regression

As shown in **Table 3**, seven of nine potential risk factors had a statistically significant association with increased odds of AHC in an unadjusted logistic regression analysis. Those with the strongest associations included direct physical contact with AHC patients (cOR: 7.74, 95% CI: 3.70–16.30, *P* < 0.001), frequent eye rubbing (cOR: 6.64, 95% CI: 3.20–13.70, *P* < 0.001) and poor hand hygiene (cOR: 5.10, 95% CI: 2.50–11.30, *P* < 0.001). Using the same toilet and sharing a towel were not found to be significantly associated with AHC in the univariable models.

**Table 3:** Crude and adjusted odds ratios for the association between behavioural risk factor and acute haemorrhagic conjunctivitis for cases and controls (*n* = 146), Pak Touch village, Cambodia, 2023

Variable	Crude OR (95% CI)	*P*	Adjusted OR (95% CI)	*P*
**Age group (years)**				
** < 6**	**Reference**	**-**	**Reference**	**-**
**6–18**	**2.85 (0.70–11.00)**	**0.129**	**2.91 (0.56–15.03)**	**0.201**
**19–30**	**1.48 (0.34–6.42)**	**0.600**	**4.80 (0.70–22.30)**	**0.112**
**31–50**	**1.47 (0.34–6.42)**	**0.586**	**4.57 (0.80–26.80)**	**0.092**
** ≥ 51**	**1.28 (0.30–5.50)**	**0.737**	**2.5 (0.40–13.90)**	**0.347**
**Sex**				
**Male**	**Reference**	**-**	**-**	**-**
**Female**	**1.05 (0.50–2.00)**	**0.868**	**–**	**–**
**Met with AHC patient**				
**No**	**Reference**	**-**	**Reference**	**-**
**Yes**	**4.19 (2.10–8.40)**	** < 0.001**	**1.30 (0.30–5.55)**	**0.738**
**Physical contact with AHC patient**				
**No**	**Reference**	**-**	**Reference**	**-**
**Yes**	**7.74 (3.70–16.30)**	** < 0.001**	**4.42 (1.90–10.10)**	** < 0.001**
**Had AHC patient in family**				
**No**	**Reference**	**-**	**Reference**	**-**
**Yes**	**3.35 (1.70–6.70)**	**0.001**	**1.05 (0.15–7.10)**	**0.957**
**Used the same toilet**				
**No**	**Reference**	**-**	**-**	**-**
**Yes**	**1.40 (0.70–2.70)**	**0.318**	**–**	**–**
**Shared eye drops**				
**No**	**Reference**	**-**	**Reference**	**-**
**Yes**	**2.90 (1.26–6.65)**	**0.012**	**2.50 (0.90–7.20)**	**0.083**
**Shared cups for drinking**				
**No**	**Reference**	**-**	**Reference**	**-**
**Yes**	**2.90 (1.50–5.70)**	**0.002**	**1.30 (0.50–3.10)**	**0.570**
**Shared towel**				
**No**	**Reference**	**-**	**-**	**-**
**Yes**	**1.50 (0.70–3.00)**	**0.280**	**–**	**–**
**Rubbed eyes frequently**				
**No**	**Reference**	**-**	**Reference**	**-**
**Yes**	**6.64 (3.20–13.70)**	** < 0.001**	**4.56 (2.00–10.37)**	** < 0.001**
**Poor hand hygiene**				
**No**	**Reference**	**-**	**Reference**	**-**
**Yes**	**5.10 (2.50–11.30)**	** < 0.001**	**3.70 (1.64–8.43)**	**0.002**

AHC: acute haemorrhagic conjunctivitis; CI: confidence interval; OR: odds ratio.

Bold *P*-values are statistically significant (< 0.05).

The three factors with the highest crude ORs remained significantly associated with increased odds of an AHC infection in the multivariable analysis (**Table 3**). Having physical contact with an AHC patient and frequent eye rubbing both increased the odds of AHC more than fourfold (aOR: 4.42, 95% CI: 1.90–10.10, *P* < 0.001; aOR: 4.56, 95% CI: 2.00–10.37, *P* < 0.001, respectively). Poor hand hygiene was also independently associated with AHC infection (aOR: 3.70, 95% CI: 1.64–8.43, *P* = 0.002). All other factors including meeting with an AHC patient, having an AHC patient in the family, and the other sharing and hygiene-related behaviours were not significantly associated with infection after adjusting for confounders.

### Laboratory findings

Among the 21 conjunctival swabs tested, real-time RT–PCR detected enterovirus in 11 samples. Nine of these showed coinfection with rhinovirus, and five were also positive for human coronavirus NL63 (HCoV NL63). Further RNA sequencing of five samples that were positive for enterovirus revealed the highest similarity to CV-A24v, confirming it as the cause of the AHC outbreak.

## Discussion

AHC, or “pink eye,” a highly contagious viral infection with a short incubation period, has caused several global outbreaks over the past four decades. In recent years, most notably in 2023, large-scale epidemics occurred across Asia, mainly due to infection with CV-A24v. Because of its high infectivity, AHC remains a significant public health risk, with potential medical, social and economic impacts. (*28*, *29*) Recent outbreaks highlight the need for robust surveillance and targeted response strategies.

Enterovirus infections can occur year-round, but tend to peak in spring and autumn. (*30*) In the autumn of 2023, India, Nepal, Pakistan and Viet Nam all reported surges in cases of infectious conjunctivitis. (*31*) Multiple outbreaks also occurred during Thailand’s rainy season (September–October). (*32*) Thus, Cambodia's spike in AHC cases in September 2023, at the end of its rainy season, is unsurprising. However, it was Cambodia’s first outbreak of AHC since 1980. (*26*) This does not necessarily mean that such outbreaks have not occurred during the past 43 years. The lack of investigations and limited emphasis on surveillance for eye health may have led to cases being overlooked or misidentified, suggesting that the extent of the presence of AHC in the country may have been underreported.

The wide age range of cases (1–70 years) and the lack of a significant gender predisposition highlight the disease’s ability to affect people of all ages and genders. However, this analysis showed that individuals aged 6–18 years accounted for 26% of cases, a proportion similar to that in those aged 31–50 years. While this does not confirm increased susceptibility, the involvement of school-aged children may reflect exposure patterns associated with communal settings, such as schools and social gatherings. Similar trends were observed in a 1972 outbreak in Tunisia and a July 2023 outbreak in India, both of which saw a higher proportion of cases among school-aged children, although older individuals were also affected. (*33*, *34*)

This retrospective case-control study identified several key risk factors for AHC transmission including physical contact with an AHC patient, frequent eye rubbing and poor hand hygiene. Similar risk factors have been reported in previous outbreaks, including the 1985 outbreak in Singapore and the 2003 outbreak in Melaka, Malaysia, in both of which close contact and the sharing of personal items played major roles in transmission. (*6*, *35*) These findings underscore the importance of focusing on personal hygiene and avoiding sharing items as a basis for developing control and prevention strategies.

Immediately following the initial investigation, the Ministry of Health, in collaboration with the National Program for Eye Health, initiated a comprehensive public health education campaign to alert the public to the symptoms and modes of transmission of and preventive measures for AHC, such as practising good hand hygiene and avoiding contact with infected individuals. The public health education programme targeted schools, health-care facilities and community leaders, and used multiple communication channels, including local media and social media platforms, to deliver its messaging.

The propagated epidemic curve in this study showed a rapid increase in cases within 1 week, reflecting the characteristically swift transmission of AHC during outbreaks. Clinically, the individuals affected by the outbreak presented with typical signs, such as conjunctival hyperaemia, subconjunctival haemorrhage, discharge, eyelid swelling, pain and tearing. The high prevalences of subconjunctival haemorrhage (82.2%) and conjunctival hyperaemia (100%) highlight the distinct and severe presentation of AHC compared with other types of conjunctivitis. In most respects, clinical manifestations were similar to those reported in the 2023 outbreak in India, in which conjunctival hyperaemia was also observed in all cases. (*34*) However, this study observed higher rates of subconjunctival haemorrhage than in outbreaks in Viet Nam in 2023 and India in 2022, which reported prevalences of 4% and 17%, respectively. (*30*, *31*) The high prevalence of subconjunctival haemorrhage in the cases in this study might be linked to a high prevalence of frequent eye rubbing, which can cause subconjunctival haemorrhage secondary to AHC. Other signs, such as eyelid swelling, tearing, pain and discharge, had prevalences comparable to those in both earlier and more recent investigations. (*34*, *36*)

The AHC surge in Pak Touch village began with the four cases observed among eight individuals who returned home from Viet Nam on 14 September 2023. Therefore, it is possible that the Pak Touch outbreak may be linked to the outbreak of CV-A24v infection in Viet Nam in 2023, as reported by Tran et al. (*31*) News reports from early September 2023 indicated that the Viet Nam outbreak may well have contributed to the rise in AHC cases observed in Cambodia, but further investigation would be needed to confirm this. (*37*, *38*) Nevertheless, these events highlight how quickly diseases can spread globally and underscore the need for effective surveillance systems to detect and respond to outbreaks promptly. Consequently, international coordination is crucial for sharing information and implementing unified public health strategies to control cross-border infections.

The advanced PCR methods used in this study allowed for rapid identification of the virus and revealed the presence of enterovirus in 11 of 21 conjunctival swab samples. Interestingly, nine of these positive samples showed coinfection with rhinovirus, and five had additional coinfection with HCoV NL63. These findings are consistent with those from a study conducted in Madurai, India, in 2021 during the COVID-19 Delta surge, during which many AHC cases caused by CV-A24v infection also involved coinfections with *Human adenovirus D* (HAdV-D) and severe acute respiratory syndrome coronavirus 2 (SARS-CoV-2). (*39*) Similarly, the 2023 AHC outbreak in Viet Nam, which was linked to CV-A24v, revealed coinfections, including three cases also with HAdV-D, two with herpes simplex virus type 1, and one each with Epstein–Barr virus and cytomegalovirus. (*31*) This similarity highlights the recurring nature of coinfections during viral outbreaks and underscores the importance of implementing comprehensive viral screening to manage and understand AHC. The first-line test for virological confirmation is the real-time RT–PCR assay, as it can yield results in a few hours. (*22*) However, due to a lack of sensitivity, more advanced gene sequencing techniques, such as RNA sequencing with next-generation sequencing, are required to fully characterize the responsible pathogen. (*22*) Although considered the gold standard for virus isolation, (*22*) these methods pose challenges for timely diagnosis, particularly in resource-limited settings such as Cambodia. In this study, the use of unbiased RNA sequencing analysis confirmed the presence of CV-A24v in all five cases who tested positive for enterovirus, but it took approximately 16 days to yield definitive results.

Effectively managing AHC outbreaks in resource-limited settings such as Cambodia requires addressing several public health challenges. Currently, eye health diseases are not included in Cambodia’s case-based, event-based and sentinel surveillance systems, which often leads to delayed reporting. (*40*) However, while extending these systems to remote areas, such as Pak Touch village, may be desirable, it may be difficult for systems that rely on digital tools – such as CamEwarn (a case-based surveillance system for seven epidemic-prone diseases) and Hotline-115 (for reporting diseases) – because local health centres often lack the necessary computers, reliable internet connections and technical skills. A more feasible way forward may be to implement enhanced community-based syndromic surveillance, which would require training local health-care workers and volunteers to recognize early symptoms of AHC and to report via a mobile app or SMS integrated with Hotline-115.

### Limitations

This study had several limitations. Its retrospective design and reliance on historical and self-reported data risk introducing bias due to recall inaccuracies and missing or incomplete records. The epidemic curve, for example, was based on patients’ recollections of the date of symptom onset, which may have affected the accuracy of the outbreak timeline. Data collection ceased on 24 September, potentially resulting in missed cases and an underestimation of the true attack rate. The small number of conjunctival swabs and samples for sequencing limited the ability to draw more robust conclusions about the viral strains and their transmission dynamics. Furthermore, the lack of phylogenetic analysis limited insights into the genetic diversity and evolutionary changes of the virus. Finally, the findings from the risk factor analysis may not be generalizable to other areas, given the specific sociodemographic and environmental characteristics of Pak Touch village.

### Conclusions

The AHC outbreak in Pak Touch village, Ratanakiri province, Cambodia, was attributed to infection with CV-A24v. Critical risk factors for infection in this setting included physical contact with AHC patients, frequent eye rubbing and poor hand hygiene. These findings are essential for developing targeted public health interventions to control and prevent future outbreaks of AHC in Cambodia. Continual surveillance and public education remain crucial to manage AHC and reduce its public health impact in the future.
